# Challenges of Recruiting Patients Into Group-Based Stroke Rehabilitation Research: Reflections on Clinician Equipoise Within the Singing for People With Aphasia (SPA) Pilot Trial

**DOI:** 10.3389/fpsyg.2021.624952

**Published:** 2021-06-03

**Authors:** Raff Calitri, Mary Carter, Chris Code, Ruth Lamont, Sarah Dean, Mark Tarrant

**Affiliations:** ^1^Institute of Health Research, University of Exeter Medical School, University of Exeter, Exeter, United Kingdom; ^2^Department of Psychology, University of Exeter, Exeter, United Kingdom

**Keywords:** equipoise, singing group intervention, recruitment, clinical trial, aphasia

Recruitment into clinical trials is a major challenge (McDonald et al., 2006; Sully et al., 2013; Amstutz et al., 2017; Walters et al., 2017). Recruitment that is too slow can lead to trials being halted and new, promising interventions abandoned (Amstutz et al., 2017). This has the concomitant effects of wasting participants' time, research funding, and clinical resource, none of which sits well ethically. Where clinical populations are the focus of trials (such as a physical rehabilitation intervention for stroke survivors, or an online CBT programme for anxiety and depression), multiple sources are often utilized to recruit participants into research (e.g., patient lists, community groups, media-advertisements, disease-specific support programmes). Clinicians themselves often adopt a “gatekeeper” role, ultimately deciding whether or not to recommend a trial to their patients. Clinicians can therefore play a fundamental role in the success or failure of recruitment.

In this paper, we reflect on our experiences of the recruitment process during the recently-reported Singing for People with Aphasia (SPA) pilot feasibility Randomized Controlled Trial (RCT) (Tarrant et al., 2018). The project was completed successfully (Tarrant et al., 2021) and there was overwhelming support and positivity from clinicians (Speech and Language Therapists; SLTs), without which the study would not have succeeded. However, there were some challenges which considerably slowed participant recruitment. These issues will be of interest to behavioral scientists, trial managers, and other researchers who need to work with clinicians when recruiting to RCTs.

## Overview of the Spa Trial

The SPA intervention was a 10-week community-based singing group programme designed for adults with post-stroke aphasia. It was led by a trained singing facilitator and co-facilitated by a “singing champion,” who themselves had post-stroke aphasia. The intervention drew upon key principles of the Social Identity Approach to Health (Jetten et al., 2017) and the Social Identity Model of Behavior Change (Tarrant et al., 2020). A key premise of these theoretical positions is that an individual's sense of psychological connection and embodiment of the treatment group affords an opportunity for social support and related group processes which provide a protective buffer against decrements in well-being or even potentially increases well-being. In other words, a positive and strong sense of group bonding (social identity) with others in the treatment group may benefit individuals in ways that promote well-being. This could translate into improvements in speech and language through encouragement, confidence, efficacy, and trust within the group environment. Past research has established that singing groups quickly harness social identity processes through co-ordinated, shared experiences (Pearce et al., 2015; Tarrant et al., 2016). The SPA intervention explicitly articulated how to optimize social connections within a singing group.

The aim of the SPA pilot trial was to assess the acceptability and feasibility of the trial processes as well as the intervention itself. The trial informed the decision on whether a definitive RCT, assessing the clinical and cost effectiveness of SPA would be possible (Tarrant et al., 2021). A key consideration was our ability to recruit the target number of participants within 6 months. The target sample size was 48 participants with post stroke aphasia and the achieved sample size was 41. This sample size was reached after 11 months and only following an amendment to the protocol to run the study in an additional location. Concern about the lack of engagement from some SLTs was highlighted through this process.

### Clinician Engagement

SLTs within early supported discharge (community facing) teams across two counties of the UK were approached by the study team and asked to support recruitment. The SLTs expressed support for SPA and believed strongly that the intervention would benefit their patients. However, as the recruitment progressed, it became clear that this belief did not always translate into “referrals” or recommendations. In fact, following discussion with some SLT leads, it became clear that precisely *because* they viewed the intervention as beneficial they were not prepared to deny their patients access to it. From the perspective of these clinicians, the random allocation of participants to the intervention (the SPA programme + a resource pack) or the control group (resource pack alone), would mean that around half of those recruited *would not* get access to the singing group. In addition, these same participants would have to forgo joining any other singing or lifestyle interventions until after the trial had ended around 6 months later (a participation requirement). Reluctance to recommend patients into a trial is by no means unique to SLTs: similar unwillingness has been reported in oncologists who refused patients entry into a new RCT of lung metastasectomy, unprepared to deny vulnerable patients an assumed beneficial treatment option (Treasure et al., 2020).

## Putting Clinical Engagement in Context

The SLT's belief that SPA would be of benefit was likely influenced by the recent growth of singing groups across the UK, and also anecdotal evidence of improvements in well-being and health symptoms from stroke patients who have participated in them. Research teams clearly need to be aware of this position and the tension it presents. Clinicians are burdened with putting patient health and well-being first and so, when balancing the harms and benefits of new interventions or therapies, are justifiably biased in their priorities. So, when faced with promising treatments, clinicians would not want to deny their patients the opportunity of receiving it. In the context of SPA, the denial of access to an intervention was seemingly justifiable on the grounds that they did not wish half of the patients to miss out—they had heard only good things about existing community singing groups, which they equated with SPA, and therefore it seemed reasonable that their patients should make the most of the services available locally (and not sign-up to the trial). This is a valid position. However, setting aside for a moment the equation of SPA and other community singing groups (we return to this later under “lessons learned”), we must remember that from a scientific perspective existing community singing groups have also not yet been submitted to a fair test of their effectiveness. There is no robust evidence attesting to the therapeutic utility of the singing groups or indeed any evidence that rules out potential harms. Put simply, we do not actually know whether or not existing singing groups for aphasia are effective for improving well-being, speech, or language.

## (No) Equipoise

The reluctance of some SLTs to offer the SPA trial to their patients is understandable, but this lack of engagement is problematic for RCTs as is creates a problem with equipoise. In order to conduct a fair test of a new intervention, it is important, at theoretical and practical levels, that all those involved remain in a position of balance, known as “equipoise,” which means being uncertain about whether the intervention will work or not. In reality, however, the position of equipoise is not straightforward. The requirement to be in equipoise is conceptually challenging when so much time has already been invested in getting a new intervention ready to be tested in a trial. It can also be practically difficult to be in equipoise when interacting with patients: there has been debate over whether clinical responsibility for patient welfare can be maintained under the conditions of clinical trials, where some patients may be given inferior treatment when allocated to a clinically inactive (control) trial arm (Miller and Joffe, 2011; Rabinstein et al., 2016; Hey et al., 2017). In the case of SPA, there is no definitive evidence that singing groups “work.” Clinicians should not have found themselves in a position where their clinical responsibilities for patient welfare conflicted with or were jeopardized by the patient's participation in the RCT. Nevertheless, there was an apparent strong belief that the control group amounted to the provision of inferior care. We discuss below how the research team could have dealt with this better.

While equipoise in clinicians is of importance for providing a fair test of an intervention, recent research highlights that it is an important position that needs to be adopted by all parties involved in RCTs, including patients, researchers and clinicians (Norris et al., 2019; Lehmann et al., 2020; Sherratt et al., 2020). Reduced quality and a biased trial await if all parties do not engage from a position of equipoise but instead hold strong opinions about an intervention's (as yet untested) utility and effectiveness. Clinicians, therefore, can best support research and the effective use of interventions by approaching RCTs with an open mind and ensuring that patients are given opportunities to participate. How they communicate this information to their patients, which will help patients themselves to make an informed decision about their participation, is vital. Indeed, failing to engage patients provides a second source of bias—removing patient choice.

## Denial of Patient Choice

Denying patients an opportunity to participate in research is one consequence of clinicians not engaging or not being in a position of equipoise—akin to a muting of the patient's voice. Patient-centered care is strongly advocated in the National Health Service, with clinicians encouraged to include patients in the decision making around their care by providing treatment options and clear information to help them make choices (National Health Service, 2005). Patient-centered care has had a positive impact, with this model of care shown to improve adherence to treatments (Thompson and McCabe, 2012) and lead to better health outcomes (Ekman et al., 2012). A patient-centered approach to engaging patients in *research* is also considered good practice (The Lancet, 2005; Evans et al., 2016). However, there is still work to be done (Sacristán et al., 2016). Patients should be offered the opportunity to take part in research and provided with all necessary information so that they can make a fully informed decision. Even though clinicians may be concerned about patients not necessarily getting direct benefit from participation in an RCT, this stance is not always shared by patients. Patients understand the importance of research and many report being prepared to forgo active treatment (i.e., being allocated to the control group) in order to support the “greater good,” recognizing they can make valuable contributions to work that may help others suffering from the same condition as them (Norris et al., 2018; Tarrant et al., 2021).

### Lessons Learned

This article is a reflective exercise but is intended as a reminder that for clinical trials to be given a “fair test” of effectiveness, it is essential that all those involved—patients, clinicians, commissioners, researchers—need to remain in a position of equipoise. Research teams should not be inactive in this process. There is great responsibility for researchers to engage sensitively with clinicians and hold a magnifying glass against all potential sources of bias, including their own. With SPA, we could have more strongly reminded clinicians that there is a lack of definitive evidence about the effectiveness of singing groups in general, and SPA in particular, and also that patients are entitled to be given choice in both their treatment and how they support research. However, we must also take the perspective of the clinician. Putting the issues above to one side, SLTs were presented with an intervention that was essentially seen as “another singing group” and perhaps not unique from existing community singing groups. This might be an inherent problem for group-based interventions like SPA that focus on cultivating meaningful social connections between recipients. To attendees or onlookers, these interventions may look like any other of the widely used community support groups, so why would they need to be shown, empirically, to “work”?

To overcome this, research teams must clearly articulate how their interventions are distinct from existing groups that may/may not be evidence-based and outline the risks/benefits it may have over its predecessors. In the same vein, the researchers could have more clearly articulated the necessity for asking all study participants (both intervention and control) not to join other singing groups and made clear that missing out on enjoyable and social gatherings (in existing community singing groups) is not necessarily comparable to missing out on a potentially effective treatment (as with the intervention singing group). SLTs could have then given their patients the option to make an informed decision to take part. Perhaps, from the SLT's perspective, SPA did not do this well enough at the time of the trial. The SLTs may not have perceived the significance of evidencing the health benefits of “groups” generally, and singing groups specifically.

Despite the challenge we faced from some SLTs, it is important to reiterate that there was very strong support for the research and engagement overall. Whilst we did not quite reach our recruitment target, the help we received from SLTs was highly valued and essential to the success of SPA. Our reflections on the challenges faced are utilitarian and provide an opportunity for us to refine and improve our engagement with clinicians as we work toward a definitive trial of SPA. We hope that these learnings might also alert researchers conducting applied group-based research to potential recruitment challenges when working with clinical populations and equip them with the necessary foresight to prepare to work with clinicians to recruit participants.

## Author Contributions

RC conceived this opinion piece with MT. RC and MC worked with speech and language therapists during recruitment to the SPA trial and maintained notes on SLT engagement, which are reflected here. RL, SD, and CC are part of the SPA team and provided input on the direction and tone of the article. RC wrote the first draft. MC, CC, RL, SD, and MT commented on each draft. All authors contributed to the article and approved the submitted version.

## Conflict of Interest

The authors declare that the research was conducted in the absence of any commercial or financial relationships that could be construed as a potential conflict of interest.
